# Molecular Dynamics Simulations for Resolving Scaling Laws of Polyethylene Melts

**DOI:** 10.3390/polym9010024

**Published:** 2017-01-12

**Authors:** Kazuaki Z. Takahashi, Ryuto Nishimura, Kenji Yasuoka, Yuichi Masubuchi

**Affiliations:** 1 Multi-Scale Soft-Matter Simulation Team, Research Center for Computational Design of Advanced Functional Materials, National Institute of Advanced Industrial Science and Technology (AIST), Central 2, 1-1-1 Umezono, Tsukuba, Ibaraki 305-8568, Japan; 2 Department of Mechanical Engineering, Keio University, 3-14-1 Hiyoshi, Kohoku-ku, Yokohama, Kanagawa 223-8522, Japan; r.nishimura2016@gmail.com (R.N.); yasuoka@mech.keio.ac.jp (K.Y.); 3 National Composite Center, Nagoya University, Furocho, Chikusa, Nagoya 464-8630, Japan; mas@nuap.nagoya-u.ac.jp

**Keywords:** molecular dynamics simulations, polymer melts, scaling law

## Abstract

Long-timescale molecular dynamics simulations were performed to estimate the actual physical nature of a united-atom model of polyethylene (PE). Several scaling laws for representative polymer properties are compared to theoretical predictions. Internal structure results indicate a clear departure from theoretical predictions that assume ideal chain statics. Chain motion deviates from predictions that assume ideal motion of short chains. With regard to linear viscoelasticity, the presence or absence of entanglements strongly affects the duration of the theoretical behavior. Overall, the results indicate that Gaussian statics and dynamics are not necessarily established for real atomistic models of PE. Moreover, the actual physical nature should be carefully considered when using atomistic models for applications that expect typical polymer behaviors.

## 1. Introduction

Rheological predictions for specific polymer materials must be improved for advances in polymer-based technologies. Fundamentally, this problem originates from the complexity of polymer structure, dynamics, and physical properties. For example, processes that govern polymer properties change drastically over different time scales. Importantly, phenomena that occur over a wide range of timescales are closely related to each other; i.e., structure and dynamics at the micro-scale can affect properties at the meso- and macro-scales [1,2,3]. Many polymer models have been developed for each scale and have been studied for many years [4,5,6,7,8,9,10,11,12]. At the micro- and meso-scales, molecular dynamics (MD), Monte Carlo, and metadynamics simulations that utilize molecular models of polymers are promising approaches [13,14,15,16]. In particular, MD simulations can estimate entangled polymer dynamics via explicit equation-of-motion calculations of intra- and intermolecular interactions. Recent advances in computer power have enabled a wide range of MD simulations for polymers [17,18,19,20,21,22,23,24,25]. It is now possible that the actual physical nature of each molecular model can be precisely evaluated and discussed. The universality of polymer dynamics predicted by theoretical approaches that use single chains and mean-fields has not been established for actual molecular models. For example, Gaussian statistics assumed in Rouse models is not observed for molecular models unless the molecular weight is sufficiently high. Non-Gaussian statistics affects the dynamics, which then deviate from predictions of the Rouse model. While these deviations are often concealed in scaling laws, this issue should be carefully considered.

An all-atomistic (AA) molecular model is potentially the most precise classical model; however, the equilibrating of AA polymer systems is much more difficult than that of united-atom (UA) polymer systems. For instance, Harmandaris and Kremer reported that the dynamics of AA polystyrene (PS) systems was about 120 times slower than that of UA PS systems [26]. It implies that the precise estimation of actual physical nature for AA polymer systems is challenging, even though using the recent computational power. Therefore, UA models are widely used to perform MD simulations of polymers. For UA models of the common polymer polyethylene (PE), the anisotropic united atom (AUA) [27], optimized potentials for liquid simulations (OPLS)-UA [28] and transferable potentials for phase equilibria force field (TraPPE)-UA [29] models are widely accepted [30,31,32,33,34,35,36]. MD simulations using UA PE models have been recently performed for a wide range of polymer nanocomposites [37,38], polymer interfaces [39,40,41], ring polymers [42], the nucleation of polymer droplets [43], the Fermi–Pasta–Ulam problem in realistic systems [44,45], and a better understanding of macroscopic mechanical properties [21,46,47,48,49]. However, polymer properties at long timescales need to be carefully evaluated for validation of models. Here, we performed long-time MD simulations using the TraPPE-UA PE model. To examine the actual physical nature of the model, several scaling laws for representative polymer properties were estimated and compared to some theoretical predictions.

## 2. Methodology

MD simulations of PE melts were performed using the TraPPE-UA PE model [29]. Two different types of united atoms (${\mathrm{CH}}_{3}$ and ${\mathrm{CH}}_{2}$) were defined in a PE chain, whose non-bonded interactions were described by Lennard–Jones 12–6 potentials. All bond lengths were kept rigid using the LINCS algorithm [50], whereas a harmonic potential was used to describe bond angle bending. Standard torsional potentials were used to describe rotations along bonds in the aliphatic backbone. These dihedral potentials counted also for the 1–4 non-bonded interactions. Using this UA model, we performed atomistic MD simulations for PE melts with molecular weight, *M*, ranging from 422.8 to 2807g/mol. The molecular dynamics package GROMACS [51] was used for effective computing. The different PE systems that have been simulated are presented in Table 1. Initial well-equilibrated atomistic structures were obtained by long-time MD simulations (over 100ns) with intermittent pressure rising and temperature falling processes at constant particle number, pressure, and temperature ensembles. The equilibration of systems was confirmed from comparison with previous reports [36]. The long-time MD simulations for product runs were performed with a constant particle number, volume, and temperature ensemble using the Nosé–Hoover thermostat [52,53]. To attain the precise relaxation dynamics quickly, the density *ρ* and temperature *T* were set to $0.650\;g/{\mathrm{cm}}^{3}$ and 500K, respectively. Non-bonded interactions were cut off beyond 1.2nm. The Verlet leapfrog integrator [54] was used with three-dimensional periodic boundary conditions and a time step of 2fs. For M=422.8–983.9g/mol, a total of $5\times 10^{7}$ time steps (=100ns) of equilibrium simulations were performed for three independent initial structures. For M=1405–2106g/mol, a total of $2.5\times 10^{8}$ time steps (=500ns) in equilibrium simulations were performed for six independent initial structures. For M=2807g/mol, a total of $4\times 10^{8}$ time steps (=800ns) in equilibrium simulations were performed for six independent initial structures.

## 3. Results and Discussion

### 3.1. Static Properties

**Figure 1:**
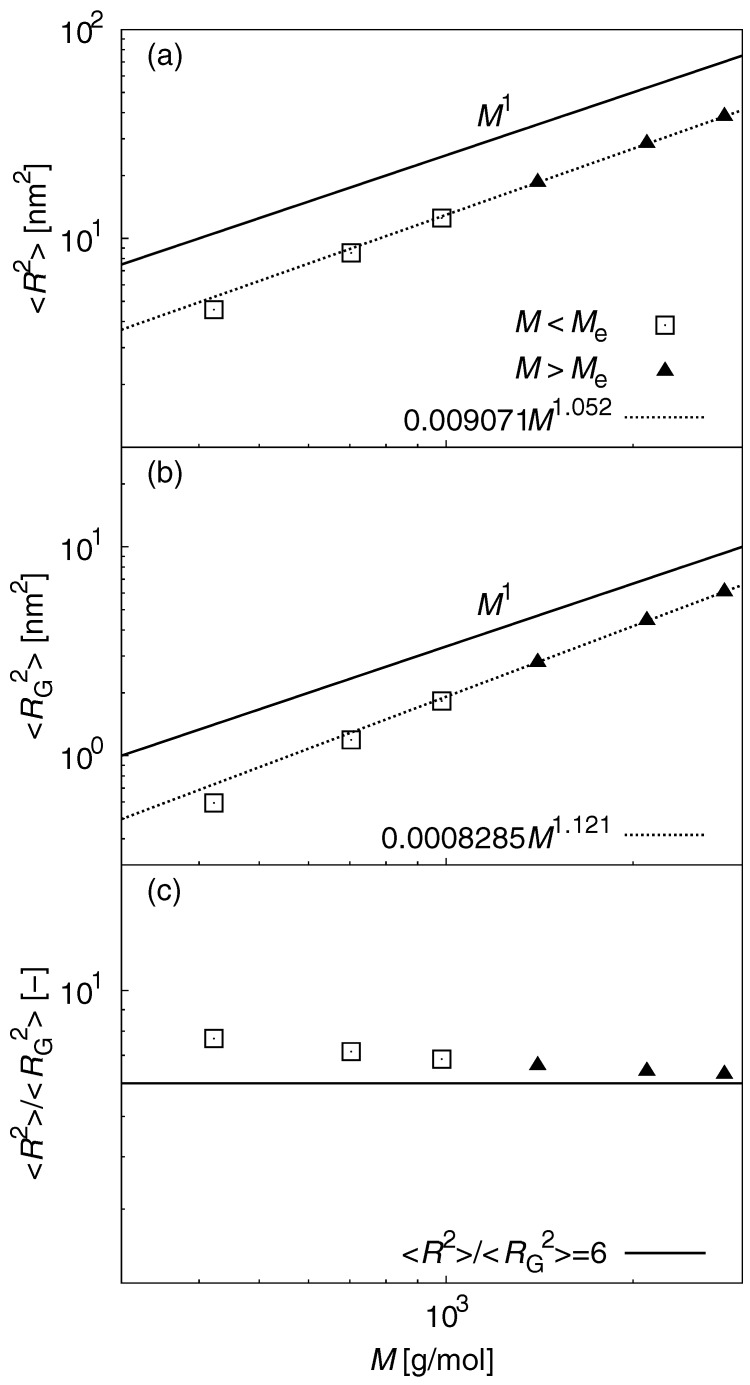
Results for (**a**) $\langle R^{2}\rangle$–*M*; (**b**) $\langle R_{G}^{2}\rangle$–*M* scalings; and (**c**) the ratio $\langle R^{2}\rangle /\langle R_{G}^{2}\rangle$ with respect to *M*. Fitting curves for data at $M>M_{e}$ are also plotted. $\langle R^{2}\rangle$ and $\langle R_{G}^{2}\rangle$ scale with $M^{1.052}$ and $M^{1.121}$, respectively. These differ by 5.2% and 12%, respectively, from values expected for ideal chains. The ratio $\langle R^{2}\rangle /\langle R_{G}^{2}\rangle$ deviates from ideal chain behavior. The slow convergence to the ideal value ($\langle R^{2}\rangle /\langle R_{G}^{2}\rangle =6$) is observed with increasing *M*.

In Flory’s theory [55] of polymer melts, equilibrium chains with uniform lengths are expected to satisfy ideal Gaussian statics. However, the chain length required to satisfy the theory is non-trivial and depends on the polymer architecture and model description. To evaluate Gaussian statics in polymer melts, the scaling law relationship between the number of beads per chain *N* (∝M). In this work, *N* is equal to the number of carbons in the PE chain), and the mean-square end-to-end distance $\langle R^{2}\rangle$, and the mean-square radius of gyration $\langle R_{G}^{2}\rangle$ were computed. The terms $\langle R^{2}\rangle$ and $\langle R_{G}^{2}\rangle$ are given by:

(1) $$\begin{array}{ccc} \langle R^{2}\rangle & = & \langle R^{2}\rangle \equiv \langle {\left(r_{N}-r_{1}\right)}^{2}\rangle , \end{array}$$

(2) $$\begin{array}{ccc} \langle R_{G}^{2}\rangle & = & \frac{1}{N}\langle \sum_{j=1}^{N}{\left(r_{j}-r_{c.m.}\right)}^{2}\rangle , \end{array}$$

(3) $$\begin{array}{ccc} r_{c.m.} & = & \frac{1}{N}\sum_{j=1}^{N}r_{j}, \end{array}$$

where ***R*** is the end-to-end vector, $r_{1}$ and $r_{N}$ are the coordinates of the chain ends, and $r_{c.m.}$ is the center-of-mass coordinate of the chain. In Flory’s theory, $\langle R^{2}\rangle$ and $\langle R_{G}^{2}\rangle$ scale as *M*. Figure 1 shows the results for (a) $\langle R^{2}\rangle$–*M*, (b) $\langle R_{G}^{2}\rangle$–*M* scalings, and (c) the ratio $\langle R^{2}\rangle /\langle R_{G}^{2}\rangle$ with respect to *M*. In general, universal polymer behavior is observed for $N>N_{e}$, where $N_{e}$ is the critical length that indicates onset of chain entanglement [1,2]. In the UA PE model, $N_{e}∼80$ was estimated from the primitive pass analysis [56,57,58]. Thus, curve fitting was done for the $\langle R^{2}\rangle$–*M* and $\langle R_{G}^{2}\rangle$–*M* at $M>M_{e}$, where $M_{e}$ corresponds to $N_{e}$. $\langle R^{2}\rangle$ and $\langle R_{G}^{2}\rangle$ scale with $M^{1.052}$ and $M^{1.121}$, respectively. These differ by 5.2% and 12%, respectively, from values expected for ideal chains. The discrepancy was observed for short-chain conditions, indicating non-Gaussian statics. The ratio $\langle R^{2}\rangle /\langle R_{G}^{2}\rangle$ deviates from the behavior of an ideal chain. The slow convergence to the ideal value ($\langle R^{2}\rangle /\langle R_{G}^{2}\rangle =6$) is observed with increasing *M*. This can be problematic when PE chains are expected to satisfy the typical polymer behavior (i.e., static universality).

**Figure 2:**
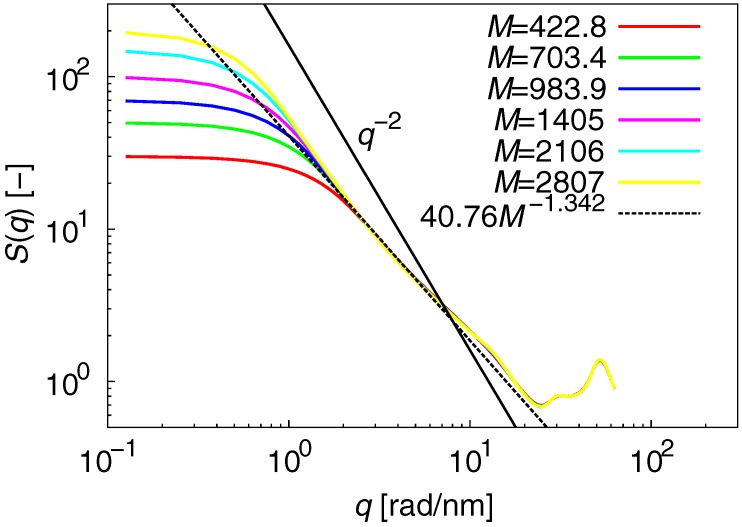
Results for S(q). The unique S(q) shape is observed for q>2.0rad/nm; however, the fractal scattering is clearly different from that expected for an ideal chain. The fractal scattering of S(q) at 2.0rad/nm<q<10rad/nm was estimated to be $q^{-1.342}$ (ν=0.7452), which differs by 33% from the expected value.

The static structure factor S(q) of an individual chain reveals the internal structure of polymer melts, and is given by:

(4) $$\begin{array}{c} S\left(q\right)=1+\frac{1}{N}\langle \sum_{j\neq k}\exp\left[-iq\cdot \left(r_{j}-r_{k}\right)\right]\rangle , \end{array}$$

where *q* is a spatial frequency equal to 2π/r, and *r* is an intra- or intermolecular distance. The fractal scattering of $S\left(q\right)∼q^{-1/\nu }$ is expected to be equal to $q^{-2}$ (ν=1/2) and be independent of chain length. Figure 2 shows the results for S(q). The unique S(q) shape is observed for q>2.0rad/nm; however, the fractal scattering is clearly different from that expected for an ideal chain. This reveals that the expected cancellation of dispersion forces for polymer melts [55] is not entirely satisfied in actual molecular models. The fractal scattering of S(q) at 2.0rad/nm<q<10rad/nm was estimated to be $q^{-1.342}$ (ν=0.7452), which differs by 33% from the expected value. These results indicate that non-Gaussian statics dominate the internal structure of polymer melts, irrespective of chain length.

**Figure 3:**
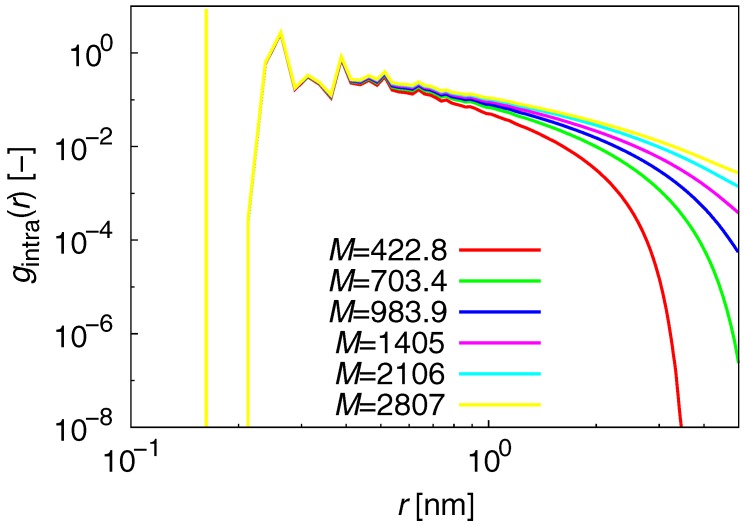
Results for the intramolecular contribution of g(r).

The radial distribution function g(r) reveals the local structure of polymer melts, and is given by:

(5) $$\begin{array}{c} g\left(r\right)=\frac{1}{4\pi r^{2}\Delta r\rho }\frac{\langle \sum_{j}n_{j}\left(r\right)\rangle }{N-1}, \end{array}$$

where $n_{j}\left(r\right)$ is the number of beads in the region between *r* and r+Δr in the molecule *j*. The term $n_{j}\left(r\right)$ can be defined for the total, intermolecular, and intramolecular contributions. Figure 3 shows the results for the intramolecular contribution of g(r), which describes the probability for beads in the same chain to meet each other. The g(r) for total and intermolecular contributions exhibit only small differences with respect to *M* (data not shown).

### 3.2. Dynamic Properties

From the Rouse [59] and reptation models [1], the scaling law relations between *N* and the end-to-end relaxation time $\tau_{R}$, and the diffusion coefficient *D*, are approximately given by:

(6) $$\begin{array}{ccc} \tau_{R} & \propto & \left\{\begin{array}{c} N^{2}\;\left(N<N_{e}\right)\\ N^{3}\;\left(N>N_{e}\right) \end{array}\right., \end{array}$$

(7) $$\begin{array}{ccc} D & \propto & \left\{\begin{array}{c} N^{-1}\;\left(N<N_{e}\right)\\ N^{-2}\;\left(N>N_{e}\right) \end{array}\right.. \end{array}$$

Evaluating whether the atomistic PE model follows Equations (6) and (7) is important for molecular modeling of polymer melts.

The term $\tau_{R}$ can be estimated from the time-correlation function of the end-to-end vector C(t):

(8) $$\begin{array}{c} C\left(t\right)=\frac{\langle R(t)\cdot R(0)\rangle }{\langle R^{2}\rangle }. \end{array}$$

**Figure 4:**
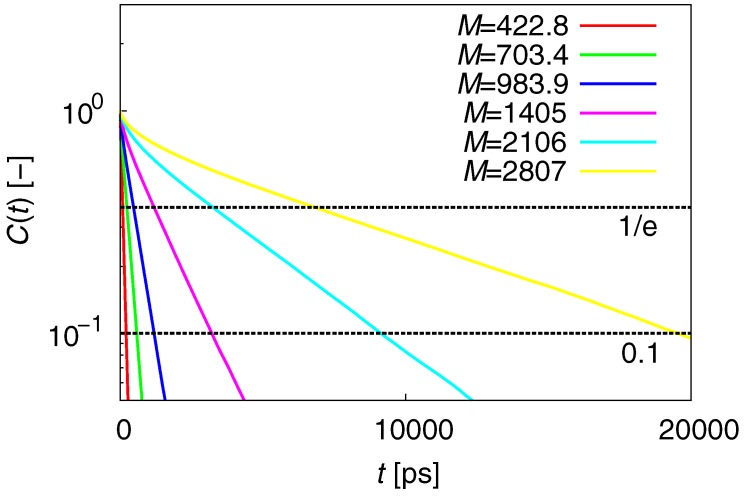
Semi-log plot of C(t). For the range 0.1<C(t)<1/e, C(t) have linear slopes, irrespective of chain length.

$C\left(t\right)∼\exp\left(-t/\tau_{R}\right)$ is expected, independent of the chain length. Figure 4 is a semi-logarithmic plot of C(t). For the range 0.1<C(t)<1/e, C(t) has linear slopes, irrespective of chain length. This indicates that C(t) clearly satisfies the above expected relation and that $\tau_{R}$ can be accurately estimated at 0.1<C(t)<1/e.

**Figure 5:**
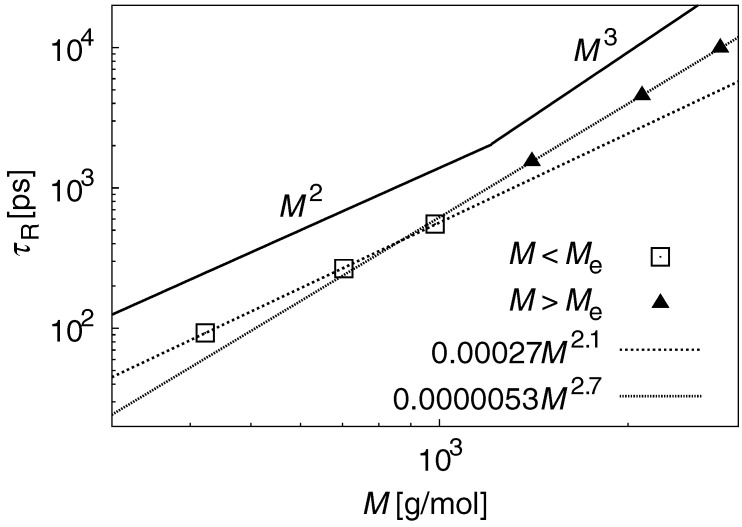
$\tau_{R}$–*M* scaling law. For $M<M_{e}$, $\tau_{R}$ scales with $M^{2.1}$, and is 5% different from the scaling exponent predicted from the Rouse theory. For $M<M_{e}$, $\tau_{R}$ scales with $M^{2.7}$ and is 10% different from the scaling exponent predicted from the reptation theory.

The term $\tau_{R}$ was estimated from C(t). Figure 5 shows the results for $\tau_{R}$–*M* scaling. For $M<M_{e}$, $\tau_{R}$ scales with $M^{2.1}$, which is 5% different from the scaling exponent predicted from the Rouse theory. This indicates that the motion of the PE chain at $M<M_{e}$ is close to ideal chain motion. For $M<M_{e}$, $\tau_{R}$ scales with $M^{2.7}$, which is 10% different from the scaling exponent predicted from the reptation theory. This indicates that the motion of the PE chain at $M>M_{e}$ is also close to ideal chain motion. However, it should be noted that a $\tau_{R}\propto M^{3.4}$ scaling relation is expected from experimental data [2].

**Figure 6:**
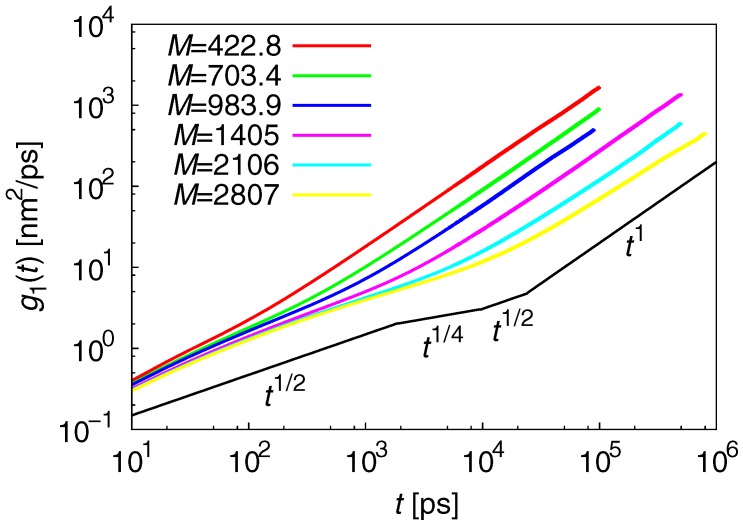
Results for $g_{1}\left(t\right)$. The expected shape of $g_{1}\left(t\right)$ from Equation (10) for M=2807g/mol is also plotted. The results of $g_{1}\left(t\right)$ at M=2807g/mol roughly have the same scaling-law, as expected. However, the threshold values for Equation (10) are unclear from the mean-square displacement results.

The term *D* can be estimated from the mean-square displacement (MSD) of the chain center $g_{1}\left(t\right)$:

(9) $$\begin{array}{c} g_{1}\left(t\right)=\langle {\left[r_{c.c.}\left(t\right)-r_{c.c.}\left(0\right)\right]}^{2}\rangle , \end{array}$$

where $r_{c.c.}$ is the coordinate of the chain center. From the Rouse and reptation models, the scaling-law sequence for the MSD is roughly expected to be:

(10) $$\begin{array}{c} g_{1}\left(t\right)∼\left\{\begin{array}{c} t^{1}\;\left(t<\tau_{0}\right)\\ t^{1/2}\;\left(\tau_{0}<t<\tau_{e}∼N_{e}^{2}\right)\\ t^{1/4}\;\left(\tau_{e}<t<\tau_{N}∼N^{2}\right)\\ t^{1/2}\;\left(\tau_{N}<t<\tau_{R}∼N^{3}/N_{e}\right)\\ t^{1}\;\left(t>\tau_{R}\right) \end{array}\right., \end{array}$$

where $\tau_{0}$ is a specific short time, and $\tau_{e}$ and $\tau_{N}$ are the Rouse relaxation times that correspond to $N_{e}$ and *N*, respectively. Figure 6 plots $g_{1}\left(t\right)$. The expected shape of $g_{1}\left(t\right)$ from Equation (10) for M=2807g/mol is also plotted. The results of $g_{1}\left(t\right)$ at M=2807g/mol have approximately the same scaling-law, as expected. However, the threshold values for Equation (10) are unclear from the MSD results.

**Figure 7:**
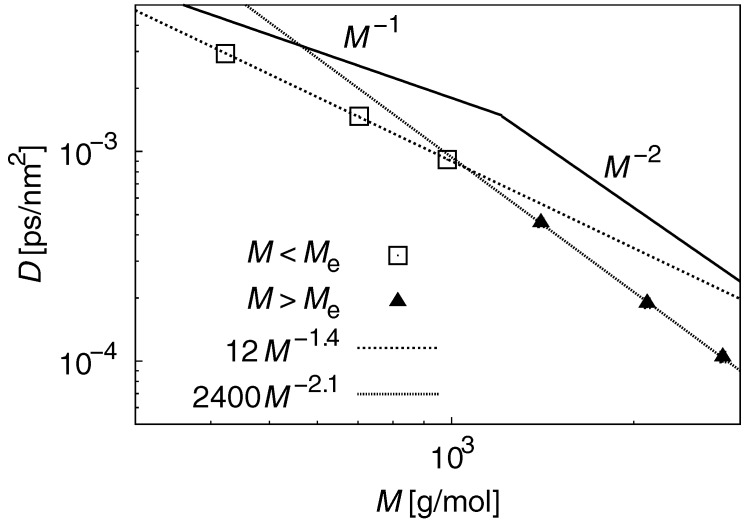
Results for the *D*–*M* scaling law. For $M<M_{e}$, $\tau_{R}$ scales with $M^{-1.4}$, while the expected value is ∼$M^{-1}$. For $M<M_{e}$, $\tau_{R}$ scales with $M^{-2.1}$, which is 5% different from the scaling exponent predicted from the reptation theory.

The term *D* was estimated from $g_{1}\left(t\right)$. Figure 7 plots the results for *D*–*M* scaling. For $M<M_{e}$, *D* scales with $M^{-1.4}$, while the expected value is ∼$M^{-1}$. This large discrepancy indicates that the motion of short chains does not reflect Gaussian dynamics and contradicts the results of $\tau_{R}$ at $M<M_{e}$ shown above. For the atomistic PE model, the relation between $\tau_{R}$ and *D* established from the Rouse theory is not satisfied. For $M<M_{e}$, $\tau_{R}$ scales with $M^{-2.1}$, and is 5% different from the scaling exponent predicted from the reptation theory. This indicates that PE chain motion at $M>M_{e}$ is close to ideal chain motion.

**Figure 8:**
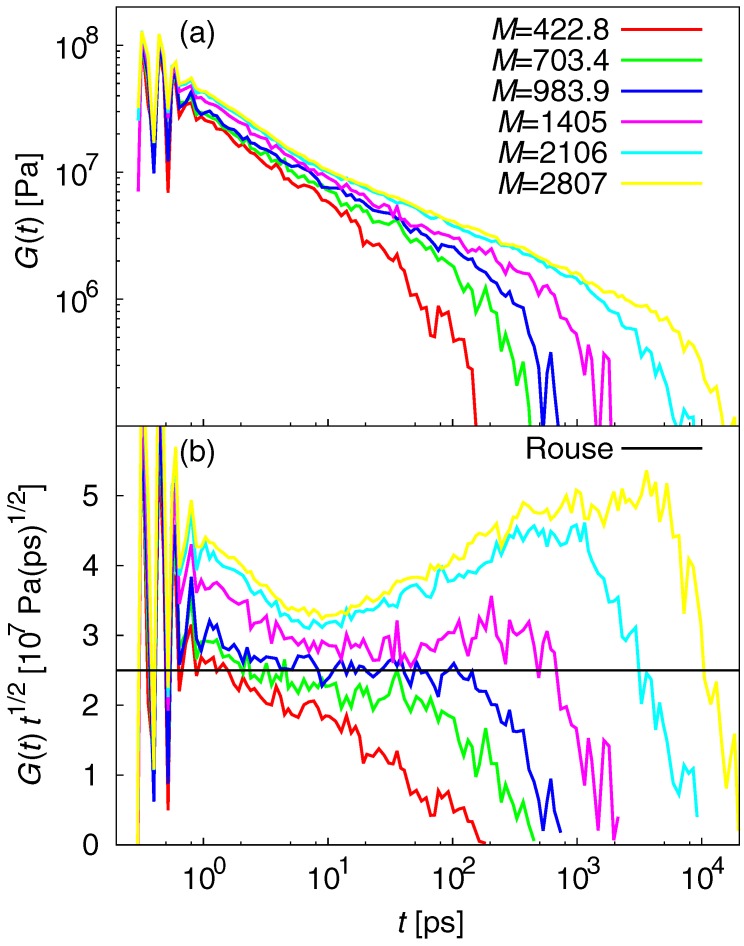
(**a**) log-log plot of G(t); and (**b**) semi-log plot of $G\left(t\right)t^{1/2}$. For M=2106 and 2807g/mol, peaks that indicate entanglement were observed at long times. Deviation from the Rouse theory that indicates onset of entanglement was observed at 10ps. The Rouse behavior can only be seen over the short-time range (5–10ps). For M=1405g/mol, the tendency is roughly the same; however, the entanglement is weak (i.e., the peak is small). For M=703.4 and 983.9g/mol, Rouse behavior is observed at 2–100ps.

The relaxation moduli G(t) reveal the viscoelastic behavior of polymer melts and are given by:

(11) $$\begin{array}{c} G\left(t\right)=\frac{V}{k_{B}T}\langle \sigma_{\alpha \beta }\left(t\right)\sigma_{\alpha \beta }\left(t\right)\rangle , \end{array}$$

where $\sigma_{\alpha \beta }$ are the off-diagonal stress components xy, xz, and yz. Figure 8a shows G(t) (log-log plot). Figure 8b plots $G\left(t\right)t^{1/2}$ (semi-log plot) to illustrate deviations from the G(t) of Rouse theory that scale as ∼$t^{-1/2}$. The semi-log plot has two advantages: (i) The *y*-axis can be compressed so that all deviations can be shown in less than one decade; and (ii) all the deviations from the Rouse theory are easily seen as deviations from the horizontal line. For M=2106 and 2807g/mol, the peaks indicate entanglement at long times. The deviation from the Rouse theory that indicates onset of entanglement was observed at 10ps. Therefore, the Rouse behavior can only be seen in the short-time range (5–10ps). For M=1405g/mol, the tendency is similar; however, the entanglement is weak (i.e., the peak is small). For M=703.4 and 983.9g/mol, the Rouse behavior is observed at 2–100ps. These results indicate that the duration of the Rouse behavior highly depends on the presence or absence of entanglement. Long-time MD simulation is a powerful way to obtain detailed results for an atomistic PE model. In contrast, the bead–spring model cannot reveal short duration Rouse behavior of entangled PE chains [60]. This illustrates the limitations of simplified models and the effectiveness of atomistic MD simulations.

## 4. Conclusions

We performed long-time MD simulations using an atomistic model of PE. To examine the actual physical nature of the model, representative polymer properties were estimated. Scaling laws were compared to theoretical predictions. For the internal structure, results for $\langle R^{2}\rangle$ and $\langle R_{G}^{2}\rangle$ indicate that the atomistic PE model for short chains does not satisfy Gaussian statics. The results for S(q) show a clear deviation from theoretical predictions that assume ideal chain statics, irrespective of chain length. With regard to chain motion, $\tau_{R}$ satisfies the prediction from the Rouse theory, while *D* clearly deviates from it. Thus, the relationship between $\tau_{R}$ and *D* in the Rouse theory is not satisfied. Regarding linear viscoelasticity, the presence or absence of entanglement strongly affects the duration of Rouse behavior. Entangled PE chains have a very short duration. These actual physical attributes should be carefully considered when using the atomistic model for applications that expect typical polymer behavior.

In general, the theoretical predictions do not reflect strictly the microscopic factors for estimating properties. For instance, the Rouse model and Flory’s theory of polymer melts rely on the single-chain motion and Gaussian statistics; however, the actual physical nature of polymers strongly affects from the many-body effect which usually cannot be expressed using the simple Gaussian statistics. This microscopic effect becomes small as the time and length scales increase but is never ignorable. MD simulations can deal with the many-body effect explicitly. This can be thought of as the main reason of the discrepancy between MD simulations and theoretical predictions.

Overall, different atomistic models may lead to different results [26,36]. The AA PE model has the potential to improve the results. The slow dynamics of AA models is a bottleneck; however, the massively parallel computing may resolve this problem.

## Figures and Tables

**Table 1:** United-atom polyethylene systems studied in the present work (ρ=0.65g/mol and *T* = 500 K).

*M* (g/mol)	No. of chains	Simulation time (ns)	No. of initial structures
422.8	1000	100	3
703.4	600	100	3
983.9	428	100	3
1405	300	500	6
2106	200	500	6
2807	150	800	6
